# Streptokinase production from *Streptococcus dysgalactiae* subsp. *equisimilis* SK-6 in the presence of surfactants, growth factors and trace elements

**DOI:** 10.1007/s13205-014-0209-x

**Published:** 2014-03-20

**Authors:** Shilpi Bhardwaj, Jayaraman Angayarkanni

**Affiliations:** 1 Department of Microbiology, Karpagam University, Coimbatore, 641021 Tamil Nadu India; 2 Department of Microbial Biotechnology, Bharathiar University, Coimbatore, 641046 Tamil Nadu India

**Keywords:** Streptokinase, *Streptococcus dysgalactiae* subsp. *equisimilis* SK-6, Surfactants, Growth factors, Trace elements

## Abstract

Streptokinase is an extracellular protein secreted by various strains of streptococci and is used clinically as an intravenous thrombolytic agent for the treatment of acute myocardial infarction. It is well established that the fibrinolytic activity of streptokinase originates in its ability to activate plasminogen. The present investigation was carried out to determine the extent of streptokinase production by *Streptococcus dysgalactiae* subsp. *equisimilis* SK-6, in the presence of surfactants, growth factors, trace elements and under the influence of different physical parameters. Mineral salt medium was supplemented with different surfactants, growth factors and trace elements. Effects of incubation period and inoculum volume were also studied. Results indicated that the streptokinase yield was higher in the presence of non-ionic surfactants, where Tween 80 supported maximum enzyme production (0.178 U/ml). Growth factors such as glycine and thiamine supplementation resulted in better enzyme production. Trace elements in the form of magnesium sulphate and ferrous sulphate when added in lesser quantity aided higher streptokinase synthesis. Enzyme production was severely affected in the presence of higher concentrations of these inorganic salts. A constant decrease in the enzyme production was observed beyond 48 h of incubation. Among the different inoculum sizes used, 1 % v/v inoculum facilitated highest streptokinase production (0.360 U/ml). The streptokinase production ability of *S. dysgalactiae* subsp. *equisimilis* SK-6 offers its potential industrial application for the manufacture of streptokinase.

## Introduction

Cardiovascular diseases are one of the biggest health concerns all over the world (Grundy et al. 1999). Among these, thrombosis is most widespread within the elderly population. The disease results from severe blood clotting, leading to the obstruction of blood circulation. In the physiological state, fibrin and platelets are utilized for clotting to prevent blood loss from injuries in a process called haemostasis (Furie and Furie 2008).

A blood clot or thrombus contains a mixture of platelets, fibrin and in some cases red blood cells and are of two types: platelet-rich arterial clots (also called white clots) formed under high shear stress, typically after rupture of an atherosclerotic plaque or other damage to the blood vessels and venous clots (also called red clots) containing red blood cells and formed under lower shear stress on the surface of a largely intact endothelium. Both these clots can be treated with anti-coagulant and anti-platelet drugs (Mackman 2012).

In order to properly terminate the haemostasis, a serine protease called plasmin digests blood clots via fibrinolysis. Plasmin deficiency may lead to thrombosis due to insufficient degradation of clots (Phan et al. 2011). The most rational treatment for acute myocardial infarction is likely to be the rapid administration of thrombolytic agents with or without procedures that produce persistent recanalization without rethrombosis (Tharwat 2006).

Among various thrombolytic agents, streptokinase is most effective in dissolving newly formed clots. Streptokinase is an enzyme produced by many strains of β-haemolytic streptococci isolated naturally from upper respiratory tract and is used to dissolve the fibrin matrix of blood clots, especially those in the arteries of the heart and lungs. Being a non-fibrin specific extracellular enzyme, it exerts its fibrinolytic action indirectly by activating the circulatory plasminogen (Banerjee et al. 2004).

Streptokinase has a molecular weight of 47 kDa and is made up of single-chain polypeptide of 414 amino acid residues. It is composed of three distinct domains, denoted as α (residues 1–150), β (residues 151–287) and γ (residues 288–414). The exponential increase in the application of streptokinase in various fields in the last few decades demands extension in both qualitative improvement and quantitative enhancement (Abdelghani et al. 2005).

In this study, we report the effect of various surfactants, growth factors, trace elements, incubation period and inoculum size on the production of streptokinase by *Streptococcus dysgalactiae* subsp. *equisimilis* SK-6.

## Materials and methods

### Chemicals and reagents

All the fine chemicals used were purchased from SRL Chemicals, India and were of the highest purity and analytical grade.

### Inoculum preparation

*Streptococcus dysgalactiae* subsp. *equisimilis* SK-6 (GenBank accession no. KF312378) was inoculated in 10 ml of mineral salt medium (MSM) (g/l: glucose, 5.0; yeast extract, 5.0; KH_2_PO_4_, 2.5; MgSO_4_·7H_2_O, 0.4; NaHCO_3_, 1.0; CH_3_COONa·3H_2_O, 1.0; FeSO_4_·7H_2_O, 0.02; MnCl_2_·4H_2_O, 0.02; pH 7.4) and incubated at 37 °C for 24 h. Following the development of turbidity, 1 % of the culture was used as inoculum (Baewald et al. 1975).

Results from our previous study showed that amending the MSM with carbon and nitrogen sources such as glucose (1.0 % w/v) and tryptone (1.5 % w/v), respectively, and an initial pH 7.0 followed by incubation at 37 °C enhanced the streptokinase production by *S. dysgalactiae* subsp. *equisimilis* SK-6 (Bhardwaj et al. 2013).

### Effect of chemical and physical parameters on streptokinase production

Effect of surfactants on streptokinase production by *S. dysgalactiae* subsp. *equisimilis* SK-6 was studied by incorporation of 0.05 % (v/v) of Tween 20, Tween 40, Tween 80, Triton X-100 and 0.05 % (w/v) sodium lauryl sulphate (SLS) and sodium deoxycholate (SDC) to the modified MSM. Growth factors like thiamine, riboflavin, nicotinic acid, tryptophan, glycine and l-lysine (0.1 % w/v) were used. Influence of MgSO_4_·7H_2_O (0.02, 0.04, 0.06, 0.08 and 0.10 % w/v) and FeSO_4_·7H_2_O (0.002, 0.004, 0.006, 0.008 and 0.010 % w/v) were studied. Effects of incubation period (24, 48, 72, 96 and 120 h) and inoculum size (0.05, 0.1, 0.3, 0.5, 1.0, 1.5 and 3.0 % v/v) were optimized.

### Streptokinase activity

Following incubation under optimized conditions, the cultures were centrifuged at 10,000*g* for 30 min. The cell-free supernatant was filtered through 0.45 μm cellulose acetate filter and the filtrate was considered as crude enzyme (Babashamsi et al. 2009).

Streptokinase activity was determined indirectly by casein digestion method, which is based on determination of the liberated tyrosine from digested casein (Mounter and Shipley 1958). One unit (U/ml) of streptokinase activity was defined as the amount of enzyme releasing 1 μmol of tyrosine equivalent/min. The soluble protein content of the enzyme sample was measured at 660 nm using a spectrophotometer. A standard curve was prepared using bovine serum albumin as standard protein (Lowry et al. 1951).

### Statistical analysis

All the optimization studies were conducted in triplicate and the data were analyzed using single factor analysis of variance (ANOVA). All the data are graphically presented as the mean ± standard deviation (SD) of triplicates (*n* = 3). ANOVA was performed using Microsoft Excel 2007. *P* values <0.05 were considered significant with a confidence limit of 95 %.

## Results and discussion

Rapid and higher enzyme production can be achieved by improvement in media composition and physical parameters (Oberoi et al. 2001). Accurate process optimization influences the activities of microorganisms and improves the production significantly, which is desirable for minimizing the processing cost. In biotechnological enzyme production processes, even small improvements have been significant for commercial success (Reddy et al. 2008).

### Effect of surfactants on streptokinase production

Surfactants have a variety of applications in microbial bioprocessing operations. Surfactants promote increased production of extracellular products through interaction with cell membrane components during the fermentation step (Rao and Satyanarayana 2003). The modulating impacts of surfactants on enzyme yield, activity, specificity and stability may thus be exploited in industrial enzymology. The general impact of surfactants in promotion of protein secretion is likely to involve interactions with the lipid components of cell membranes in a manner which facilitates secretion. Thus surfactants represent a valid option to be considered when microbial processes for production of extracellular proteins are being developed, especially where rate of protein secretion is a barrier (Singh et al. 2007).

**Fig. 1:**
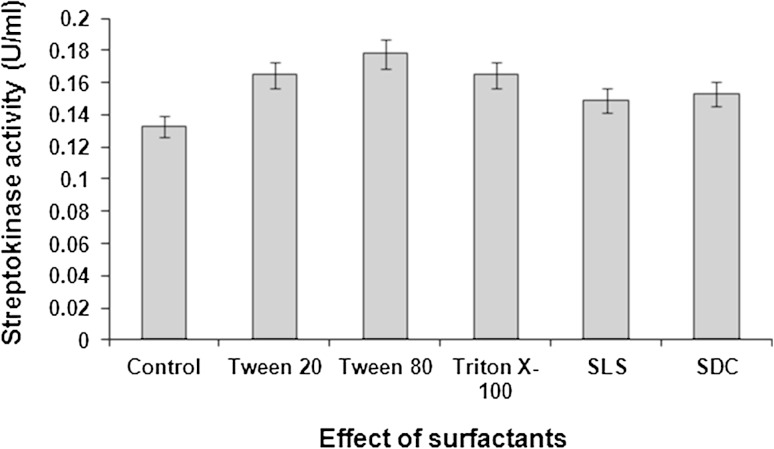
Effect of different surfactants on streptokinase production. Data represent mean ± SD (*n* = 3); *P* < 0.05

In the present study, as compared to the anionic surfactants like SLS and SDC, non-ionic surfactants such as Tween 20, Tween 80 and Triton X-100 proved to be superior in enhancing streptokinase production. Among the different surfactants used, highest streptokinase activity (0.178 U/ml) was recorded in the presence of Tween 80 (0.05 % v/v) (Fig. 1).

Tween 80 proved to be the best surfactant in *S. dysgalactiae* subsp. *equisimilis* SK-6 mediated streptokinase production possibly because of its chemical structure. Unlike other non-ionic surfactants, Tween 80 possesses greater chain length, which in turn increased its aqueous solubility thereby reducing the intensive interaction between itself and hydrophobic cell membrane. Addition of Tween 80 at a concentration of 0.01–0.10 % to the medium prior to inoculation has been recommended for achieving high titers of streptokinase which was on par with our results (Banerjee et al. 2004).

The probable reason for non-ionic surfactants enhancing streptokinase production in the present study could be due to the molecular structure of surfactants which determines varying degrees of toxicity to microbial cells. Literatures suggested that non-ionic surfactants due to their low cytotoxicity were better choices for enhancing the enzyme production. Polyoxyethylene sorbitan surfactants (Tween series) have been shown to possess low toxicity (Singh et al. 2007). Generally, surfactant toxicity becomes lower as the chain length increases, i.e. an increasing hydrophilicity (Jurado et al. 2009).

### Effect of growth factors on streptokinase production

Specific manipulations of the culture medium have been shown to enhance protein release into the medium. In the selection of a medium for economic production of streptokinase, various materials have been suggested.

**Fig. 2:**
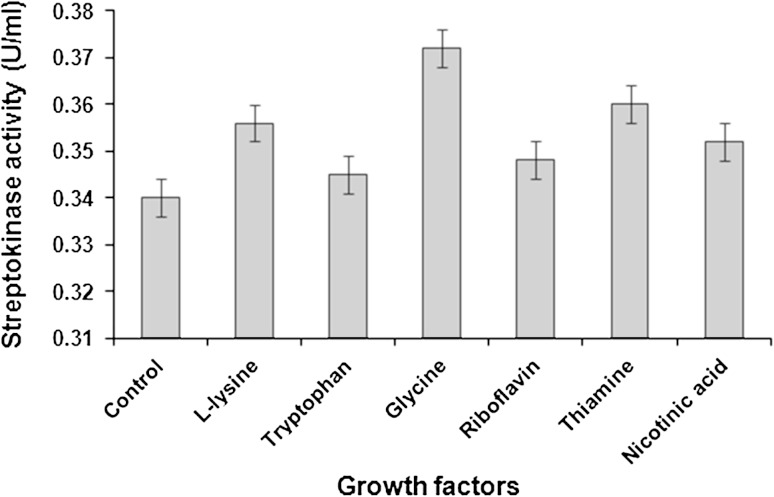
Effect of different growth factors on streptokinase production. Data represent mean ± SD (*n* = 3); *P* < 0.05

Among the amino acids, glycine supported highest streptokinase activity (0.372 U/ml) followed by l-lysine and tryptophan. As compared to vitamins like riboflavin and nicotinic acid, thiamine facilitated maximum streptokinase activity (0.360 U/ml) by *S. dysgalactiae* subsp. *equisimilis* SK-6 and was selected for further studies. Streptokinase production was greatly reduced when no growth factor was added to the medium (Fig. 2).

Glycine acted as the best amino acid probably because its supplementation enhanced the release of periplasmic proteins such as streptokinase into the medium without causing significant cell lysis (Aristidou et al. 1995).

A previous study on streptokinase fermentation reported that apart from enzyme hydrolyzed casein constituting the major part of production medium, two other ingredients, namely, glycine and an organic sulfhydryl reducing agent were also necessary. The amount of glycine employed in preparing the fermentation medium varied within wide limits, for instance, from 1.0 to 350 parts by weight of glycine for each 1,000 parts by weight of casein digest. The optimum amount had been found to be from about 200 parts of glycine for each 1,000 parts by weight of casein digest (Szumski 1962).

In general, the streptokinase fermentation medium comprised a nitrogen source such as gelatin hydrolysate or casein digest supplemented with various amino acids such as cystine, glycine, tryptophan, tyrosine, methionine and glutamine, uracil and adenine, various salts, glucose and certain members of the vitamin B group (Bernheimer and Pappenheimer 1942).

Thiamine among the vitamins supported highest streptokinase activity by *S. dysgalactiae* subsp. *equisimilis* SK-6 probably because thiamine acts as the co-enzyme thiamine pyrophosphate (TPP) in the metabolism of carbohydrates and branched-chain amino acids. Specifically the Mg^2+^-coordinated TPP participates in the formation of α-ketols as catalyzed by transketolase and in the oxidation of α-keto acids by dehydrogenase complexes (McCormick 1997). Hence, thiamine deficiency in the fermentation media might have resulted in the overall decrease of carbohydrate metabolism by *S. dysgalactiae* subsp. *equisimilis* SK-6.

In the large-scale production of streptokinase from Lancefield group of streptococci, the fermentation broth comprised growth factors such as adenine sulphate, nicotinic acid, pyridoxine, tryptophan, calcium pantothenate, thiamine HCl, riboflavin and thioglycolic acid (Ablondi and Adam 1955).

### Effect of MgSO_4_ on streptokinase production

The state of a bioprocess depends on many parameters and variables, but especially on the state of the microorganisms that are in close interaction with their micro-environment, the nature of which is the consequence of the design of the medium (Calik and Ozdamar 1999). Among the medium components, inorganic compounds can contribute to the structure of the metabolites and/or act as cofactors of the enzymes of intracellular reactions. Consequently, the essential cations in combination with their anions strongly affect bioproduct formation by influencing metabolic pathways and changing metabolic fluxes (Calik et al. 2004).

Supplementation of the production medium with MgSO_4_ had a positive influence on streptokinase production because magnesium ions play critical roles in many aspects of cellular metabolism. They stabilize structures of proteins, nucleic acids and cell membranes by binding to the macromolecule’s surface and promote specific structural or catalytic activities of proteins, enzymes, or ribozymes (Cowan 1993). It is also a key to enzymatic reactions in various ways. They can generate magnesium-substrate scaffolds to which enzymes bind. They can bind directly to enzymes and alter their structure or they may direct reactions through specific catalytic roles (Yang et al. 2004).

**Fig. 3:**
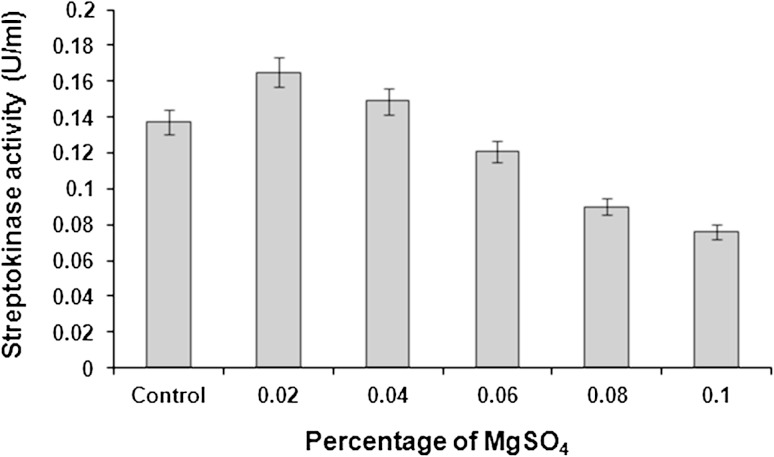
Effect of MgSO_4_ supplementation on streptokinase production. Data represent mean ± SD (*n* = 3); *P* < 0.05

Addition of different concentrations of MgSO_4_ to the MSM influenced streptokinase yield. Highest streptokinase activity (0.165 U/ml) was recorded for 0.02 % (w/v) MgSO_4_ supplementation to the medium. Increase in the concentration of MgSO_4_ beyond 0.02 % resulted in progressive decrease in the streptokinase yield. At the highest concentration of MgSO_4_ (0.1 % w/v), streptokinase production was affected severely (Fig. 3).

MgSO_4_ at specific concentrations might cause better coupling of supply and demand for the amino acids by regulation of the pathways (Calik et al. 2004). Mahmoudi et al. (2012) reported that higher level of streptokinase (580 μg/ml) was produced in the presence of MgSO_4_ (0.25 g/25 ml) as compared to the condition when no MgSO_4_ was added to the medium (360 μg/ml).

### Effect of FeSO_4_ on streptokinase production

Iron, as the ferrous or ferric ion, is essential for the life processes of all eukaryotes and most prokaryotes. However, the element is toxic when in excess of that needed for cellular homeostasis (Smith 2004). Iron is an essential transition metal in the fermentation medium. It is frequently added to the fermentation medium as a nitrate or sulphate salt. Iron complexes can form with bio-molecules such as amino acids, nucleotides, physiological chelators and proteins. The specific complexes are very important because they determine whether the iron is available to participate in cell growth and catalyze toxic reactions or becomes non-available to the system. Free or ineffectively sequestered iron can be very toxic to cells. Properly complexed iron is available to support cell life and is essential to the cell culture system.

**Fig. 4:**
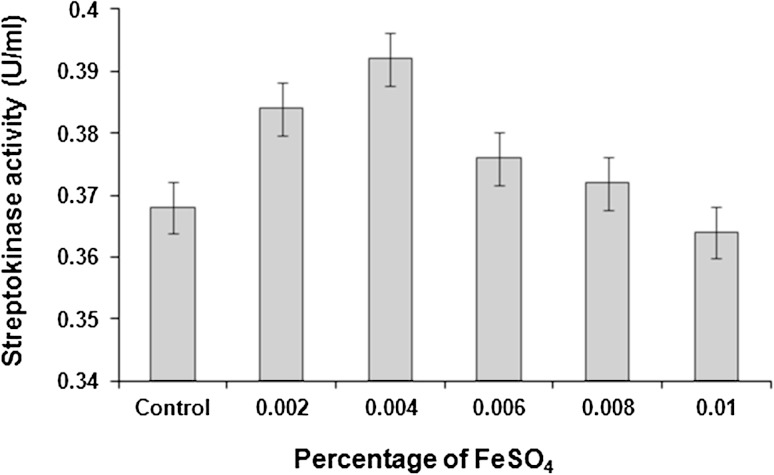
Effect of FeSO_4_ supplementation on streptokinase production. Data represent mean ± SD (*n* = 3); *P* < 0.05

Supplementation of the production medium with FeSO_4_ enhanced streptokinase production. A gradual increase in streptokinase production was observed between 0.002 and 0.004 % of FeSO_4_. Highest streptokinase activity (0.392 U/ml) was obtained with 0.004 % (w/v) FeSO_4_ supplementation to the medium. Beyond this concentration, a steady decrease in streptokinase activity was recorded (Fig. 4).

During streptokinase fermentation by *S. dysgalactiae* subsp. *equisimilis* SK-6, excess of ferrous iron in the medium could have remained unutilized and resulted in the development of oxidative stress, leading to a decrease in streptokinase production. Otherwise, excess ferrous iron might have complexed with thiols such as cysteine to form the ferrous:cysteine complex that generated hydroxyl free radicals through Fenton chemistry which could have initiated lipid peroxidation. The primary effect of lipid peroxidation is decrease in membrane fluidity, which alters membrane properties and can disrupt membrane-bound proteins significantly (Cabiscol et al. 2000).

Trace elements are usually added to the streptokinase fermentation medium in the form of a salt mixture to furnish ions of metals such as iron, magnesium, copper, zinc, etc. in very slight amounts. It is often convenient to prepare a salt mixture from the salts of these metals and add a small quantity of the mixture to each fermentation (Hawkins 1955).

### Effect of incubation period on streptokinase production

The incubation time for achieving the maximum enzyme level is governed by the characteristics of the culture and is based on growth rate and enzyme production. The enzyme production varies with incubation time (Kunamneni et al. 2005). It is very essential to detect the optimum incubation time at which an organism exhibits highest enzyme production, since organisms show considerable variation at different incubation periods (Kumar et al. 2012).

**Fig. 5:**
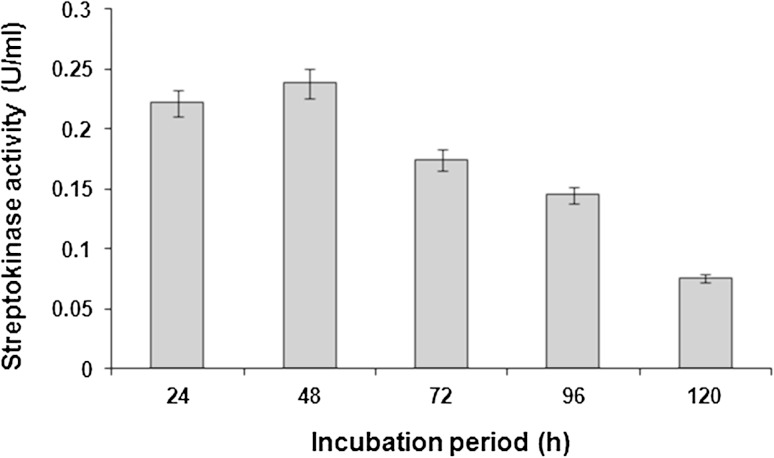
Effect of incubation period on streptokinase production. Data represent mean ± SD (*n* = 3); *P* < 0.05

Production of streptokinase from *S. dysgalactiae* subsp. *equisimilis* SK-6 was dependent on the incubation time. Streptokinase yield increased from 24 to 48 h of incubation, with the highest level of streptokinase production (0.238 U/ml) reported at 48 h. When the fermentation proceeded beyond 48 h there was a decrease in the streptokinase yield (Fig. 5).

The probable reason for decrease in the streptokinase production beyond 48 h may be due to rapid depletion of nutrients in the medium, accumulation of excess acid in the media as a result of sugar utilization and developed oxygen tension. The reduction in the streptokinase production upon prolonged incubation may be attributed to the induction of Crabtree effect. Literatures suggest that Crabtree effect leads to accumulation of acetate which is inhibitory to cell growth and generation of toxic byproducts that often limit protein yields (Ferreira et al. 2003). Lactic acid inhibition has been included in streptokinase production (Patnaik 1995).

Baewald et al. (1975) used a simple and inexpensive medium to obtain high yields of streptokinase from *S. equisimilis*. The medium contained yeast autolyzate or corn steep liquor as nitrogen source, glucose and various salts. High titres of streptokinase were attained at 28 °C, pH 7.2–7.4, within 24 h in agitated cultures. Production of streptokinase from *S. pyogenes* decreased while increasing the incubation time from 2 to 7 days (Patel et al. 2011).

### Effect of inoculum size on streptokinase production

The finite volume of a culture medium means that it can only contain limited nutrients for the microorganism. Furthermore, the consumption of the nutrients is largely dependent on the population of bacteria. To ensure a high production of enzyme in the limited volume of medium, the bacterial inoculum size should therefore be controlled (Abusham et al. 2009).

**Fig. 6:**
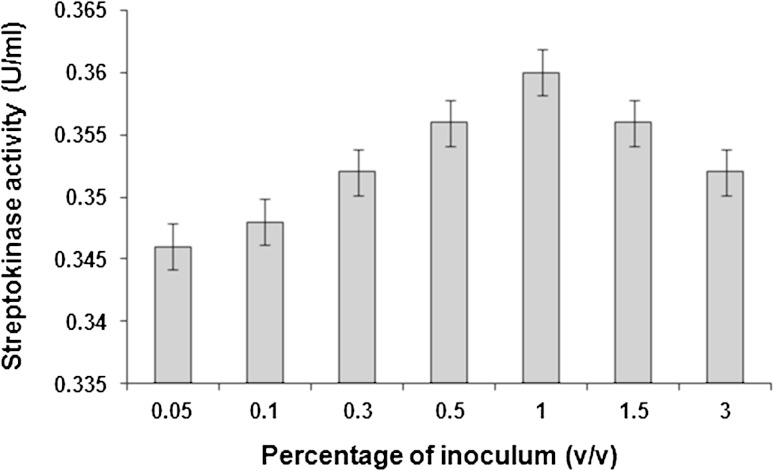
Effect of inoculum size on streptokinase production. Data represent mean ± SD (*n* = 3); *P* < 0.05

Inoculation of MSM with various inoculum sizes (0.05–3 % v/v) of *S. dysgalactiae* subsp. *equisimilis* SK-6 affected the production of streptokinase. The highest streptokinase activity (0.360 U/ml) was achieved with an inoculum size of 1 % (v/v). A higher inoculum of 3 % (v/v) was found to reduce the production of streptokinase (Fig. 6).

The increase in the production of streptokinase using small inoculum sizes could have been due to the higher surface area to volume ratio, which might have resulted in the increased production of enzyme (Rahman et al. 2005). Therefore, high inoculum sizes do not necessarily give higher streptokinase yield.

In addition, an improved distribution of dissolved oxygen and more effective uptake of nutrients might have contributed to a higher streptokinase yield. If the inoculum sizes are very small, insufficient number of bacteria would lead to a reduced amount of secreted enzyme. However, higher inoculum sizes could lead to or cause a lack of oxygen and depletion of nutrients in the culture medium (Shafee et al. 2005).

Chemically defined media for growing group A streptococci have been developed to require only small inocula and without the need for a prior adaptation regimen. The doubling times of the streptococci in such media can be comparable to those in complex media (McCoy et al. 1991). Similar to our results, Karimi et al. (2011) found that the bacterial strain *S. equisimilis* H46A (ATCC 12449) produced relatively high yields of streptokinase by the use of 1 % inoculation, pH adjustment and glucose feeding as compared to 10 % inoculation and pH adjustment. While optimizing the batch fermentation parameters involved in streptokinase production from *S. pyogenes*, it was concluded that concentration of corn steep liquor and ageing period of the producing strain were the parameters which had positive significant effect on enzyme yield while inoculum volume had negative significant effect on its yield (Patel et al. 2011).

## Conclusions

From the present study it could be inferred that streptokinase production by *S. dysgalactiae* subsp. *equisimilis* SK-6 was influenced by chemical and physical parameters such as surfactants, growth promoters, trace elements, incubation period and volume of inoculum. Incorporation of non-ionic surfactant Tween 80, vitamin, amino acid and trace elements such as thiamine, glycine, MgSO_4_ and FeSO_4_ enhanced the enzyme yield. 48 h of incubation with 1 % (v/v) inoculum size enhanced streptokinase yield. The strain *S. dysgalactiae* subsp. *equisimilis* SK-6 may be successfully utilized for streptokinase production.
